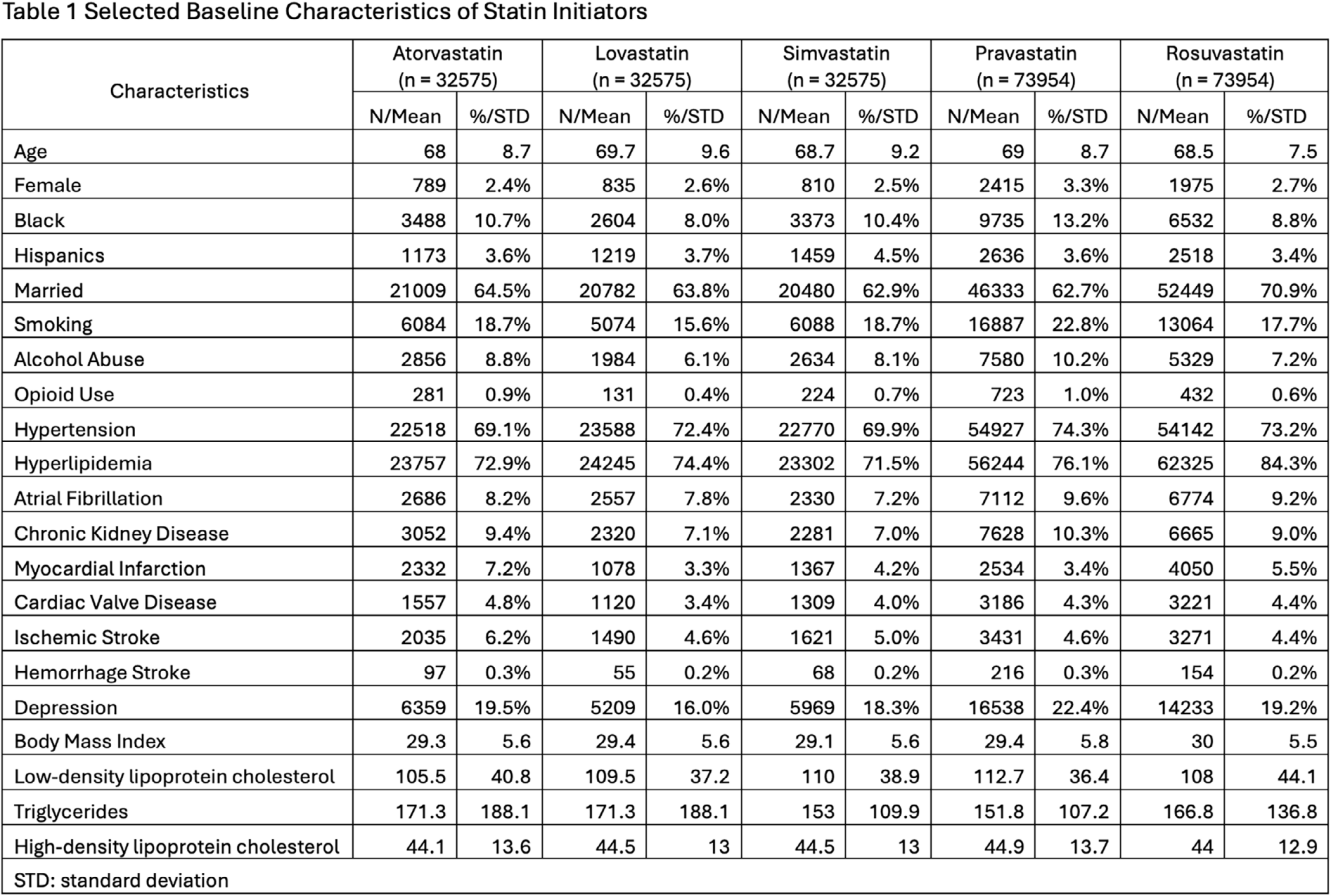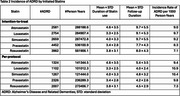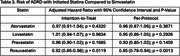# Association of Different Statins with Incident ADRD: A Target Trial Emulation Study

**DOI:** 10.1002/alz70859_107681

**Published:** 2025-12-26

**Authors:** Edward Zamrini, Yan Cheng, Ying Yin, Yijun Shao, Helen M Sheriff, Andrew Zullo, Wen‐Chih Wu, Ali Ahmed, Stuart J Nelson, Qing Zeng

**Affiliations:** ^1^ Irvine Clinical Research, Irvine, CA USA; ^2^ George Washington University, Washington, DC USA; ^3^ Washington DC VA Medical Center, Washington, DC USA; ^4^ Brown University, Providence, RI USA; ^5^ Providence VA Medical Center, Providence, RI USA; ^6^ DC VA, Washington, DC USA; ^7^ VA Washington DC Healthcare, Washington, DC USA

## Abstract

**Background:**

Statins are widely prescribed for cardiovascular disease prevention, but their association with Alzheimer’s Disease and Related Dementias (ADRD) remains unclear due to limitations in observational studies. This study emulates a target trial using real‐world data to assess the impact of different statins on ADRD risk.

**Method:**

We used the US Veterans Health Administration data to identify patients who initiated a statin between 2002 and 2021. Target trial eligibility was assessed at statin initiation based on criteria including aged 40‐100 years, no ADRD or severe psychiatric conditions, and at least two outpatient visits within two years prior. We studied both fat‐soluble statins (atorvastatin, lovastatin, simvastatin) and water‐soluble (rosuvastatin, pravastatin). To minimize bias, we sampled an equal number of initiators by total cholesterol level (unknown, <200, ≥200 mg/dl) each year for both fat‐ and water‐soluble statins. Both intention‐to‐treat (ITT) and per‐protocol (PP) approaches were used to assess the association between statin initiation and ADRD incidence. Patients were followed up to 20 years, until ADRD diagnosis, death, loss to follow‐up, whichever came first. In the PP approach, patients were also censored if they discontinued the initiated statin for more than 90 days. We fitted Cox regression models to compare the effect of different statins on incident ADRD, adjusting for baseline characteristics including demographics, comorbidities, concurrent medications, vital signs, and laboratory results.

**Result:**

A total of 245,633 patients were enrolled in the target trials (32,575 per arm of fat‐soluble statins; 73,954 per arm of water‐soluble statins), with the mean age of 68.8 years; 2.8% females, and 10.5% Blacks. The characteristics for each statin are displayed in Table 1. The mean duration of statin use ranged from 3.3 to 4.6 years, depending on the specific statin (Table 2). Rosuvastatin was associated with the lowest incidence rate of ADRD (Table 2). Adjusted Cox models also indicated that rosuvastatin was associated with a reduced risk of ADRD compared to simvastatin; however, other statins did not show a significant difference from simvastatin (Table 3).

**Conclusion:**

Our findings suggest that rosuvastatin may be superior to other statins in reducing the risk of ADRD.